# A conceptual investigation of variables affecting the success and acceptance of SMS Marketing in Iran

**Published:** 2015

**Authors:** A Adhami, A Rabiee, M Adhami

**Affiliations:** * Kerman University of Medical Sciences, Kerman, Iran; ** Payam Noor University, Tehran, Rey, Iran

**Keywords:** SMS marketing, advertising, Iran

## Abstract

This paper’s aim was to develop a conceptual overview of SMS marketing and delineate factors of new communications technologies on business practice. This study, which was a descriptive survey, was built on primary and secondary data source including a literature review of SMS marketing and a Questionnaire were used as the primary means of collecting secondary data. The sample size of 300 patients was determined according to the Cochran formula. Moreover, data analysis was done in SPSS by using linear regression, chi-square, t-test and Binomial test.

According to the research, sex, age, education, relevance, timeliness, reliability to sender, sense of control were variables affecting the SMS marketing acceptance.

This paper was qualitative and provided a solid conceptual foundation for the future empirical research on e- marketing. The potential limitation was related to the broad user of computer and mobile. In this research, we considered SMS marketing, Mobile marketing, SMS advertising as the same subject. This research will be a useful resource with important insight into the factors that may encourage or determine consumer acceptance of this new form of direct marketing.

This paper addressed an important timely issue, and added to the body of literature and knowledge focusing on e-marketing.

## Introduction and motivation

Today, the production and delivery of goods may not be sufficient because it has changed in development activities and in a competitive environment and customers must be attracted. Advertising is considered as a part of marketing success.

Successful companies, particularly in export, as well as the improvement of the quality of their products following the design, and the implementing of effective and dynamic marketing systems are highlighted.

It is perhaps not an exaggeration to assert that the mobile phone is the most ubiquitous personal item in the world. Over the years, the mobile phone has become an increasingly attractive product with added features. SMS marketing in an effort to effectively and successfully use this capacity for the introduction of the goods and services.

A long journey has been made from the radio transmitter using Morse code for maritime applications developed by Guglielmo Marconi in the 1890s, to Motorola’s “walkie-talkie” developed for the US Army during the World War II, to the current third generation of mobile telephones for the consumer [**20**].

At the outset, it is useful to define mobile marketing. In this paper, we adopted the Mobile Marketing Association’s definition: “Any form of marketing, advertising, or sales promotion activity aimed at consumers and conducted over a mobile channel” [**9**].

“Mobile marketing” is seen as the use of wireless media as an integrated content delivery and direct response vehicle within a cross-media marketing communications program.

Mobile marketing also known as wireless marketing, promises vast opportunity, still in an experimental phase business, has little experience using these new marketing tools.

However, Mobile Marketing is merely part of e-marketing. E-marketing is about much more than just the internet. It involves other technologies that enable customer relationship management, enterprise resource planning, supply chain management, text messaging, bar code scanners and digital TV [**13**].

Telephone marketing, mail and mobile telephony, as well as digital TV and the Internet, have supplemented traditional tools. In order to maximize their effectiveness, SMS, known as text messaging, is a store-and-forward communication system for the mobile phone. International Data Corporation stated that SMS is the most popular mobile data application having a recorded 65 percent of mobile phone users sending text messages daily. According to the GSM Association, cell phone users send more than 10 billion SMS messages each month, making SMS the most popular mobile data service. The use of short message service is very popular among the people of the world. According to many people, they use these services because they are “cheap” and a fast learner. Currently, 83% of English and, 79% German people use SMS. It was also anticipated that the number of messages sent in 2012 reached 3.7 trillion. This figure is double the current number of messages [**4**].

Unlike any previous technology, mobile phone is now perceived as a social necessity, especially among teenagers. The mobile phone has become a true “extension of man”.

Mobile penetration ranges from 11% for 5–8-year-olds to 81% for 13–14-year-olds and reaches saturation point at 91% for 16–18-year olds, according to the Child wise survey [**5**].

Increasingly, parents are giving children their first mobiles at the age of 8 as a way to stay in touch and for increased security and safety. Texting is as natural to children as picking up a phone. In fact, texting is overtaking speaking, particularly for girls. It is becoming their primary form of communication: 62% of the girls aged 14–16 send four or more text messages per day. They view it as more fun as well as more practical.

This massive surge in mobile ownership and usage has created a strong opportunity for brands to market especially to teens and twines in a completely new way [**6**,**10**,**12**].

Text message advertisements have been found to boost consumers’ inclination to purchase by 36 per cent, which partly explains its growing popularity among marketers. In addition, they are considered as forms of one-to-one marketing. Businesses have effectively implemented SMS advertising to update their customers, and track people and parcels [**15**].

As a media channel, the mobile is unique. While it has mass market reach, it is also a highly target able and track able interactive media. In addition, SMS advertising is also commonly used to reinforce other traditional media such as broadcast and print media. Studies have found that mobile advertising campaigns generate higher response rates as compared to direct mail and internet banner ads. Higher frequency of SMS usage shows a greater acceptance and familiarity of use of this technology [**1**,**7**,**11**,**19**].

In Iran, using short message service was put into operation for the first time in 2003. From this year onwards, the use of mobile phone text messages gradually became popular among Iranian users. In 2007, eight billion text messages were exchanged in Iran. This year, 20 million text messages were exchanged daily by mobile users, that figure was more special on days of festivities. Services SMS via GSM modem is also another way of sending bulk SMS. In this system, along with the modem, software installed on a computer and managing the SMS messages are done [**18**,**21**].

In this research, after the study and review of library literature, each of the 10 hypotheses that were based on a research question, has been tested through a questionnaire

**Research hypotheses**

1. There is no significant relationship between sex and adoption of SMS marketing.

2. The assumption is that young people are more welcoming with the SMS marketing.

3. The assumption is that individuals with a higher education are more welcoming with SMS marketing.

4. There is a relationship between the identification of the sender and the compliance with SMS marketing.

5. There is a relationship between the content of SMS marketing and the rate of acceptance.

6. There is a relationship between the timeliness of the content of the SMS marketing and the rate of acceptance.

7. Messages with customized content have more impact.

8. Messages that are sent at the appropriate time have a greater impact.

9. Repetition and frequency of SMS marketing will reduce the interest and attention of the¬ audience.

10. In the case of audiences, once they are capable of avoiding the SMS marketing, the acceptance rate will be increased.

Terminology

Sex: Is a biological concept and purpose, means woman or man

Age: Chronological age of the study participants

Personal and Institutional trust: Personal trust is composed of two components. The first component is concerned with the customer’s relationship with the company that uses SMS marketing. This relationship would be a reflection of the cumulative experiences with the company’s products and services or encounters with the company’s service personnel. This relationship shapes the customer’s perception of the company’s products and services, including its perceived trustworthiness. Personal trust can also be affected by social influence. This is based on the experiences that friends, family members, colleagues or other acquaintances in the customer’s social network have had with the company.

Related information: Get the right information to the right people.

Timely information: Access to information in appropriate time.

Personalization: Use name or title of SMS receivers to greeting them and showing understanding to their need. Sending time: when the SMS is send. Day or night.

Repetition and Frequency: Number of SMS advertising within a specified period (day).

Perceived control: Consumers perceive that they have control over the number and type of mobile messages that they receive. For example by offers an opt-out option in SMS text.

**Methodology**

Given the nature of the research objectives it was determined that qualitative research techniques would contribute to an in-depth understanding of SMS marketing, being the most appropriate for this research [**2**].

In this study, data collection methods including the searching on the Internet, studying internal and external books and journal additionally in order to achieve theoretical foundations, we took advantage of from other researcher experiences. Also a questionnaire was applied as a main tool for data collection and obtained desired data and hypothesis test.

In Iran, SMS marketing study began from 2000 by using SMS as a way of communicating. This study was run in 2013 and the questionnaire took place between Apr and May 2013.

The statistical populations are residents of Kerman. Statistical sample includes individuals who are literate in addition to using their mobile phones for the communication purposes as audio device will use to send an SMS.

In this study, the sampling was random and the Cochrane formula was used. Accordingly, the sample size was 150. But, for more accuracy in citing the research results, the sample increased to 300 people.

$$n=\frac{400000\times {\left(1.96\right)}^{2}\times 0.5(1-.5)}{{\left(0.1\right)}^{2}\times \left(400000-1\right)+{\left(1.96\right)}^{2}\times 0.5(1-.5)}\approx 150$$

The questionnaire was generated by the researcher and by the study of the research literature and marketing professors. Which is the one that includes 19 five-choice Likert scale questions?

The reliability of the test was determined by Cronbach’s alpha as it follows:

**Table 1:** Reliability Statistics

Cronbach’s Alpha	Cronbach’s Alpha Based on Standardized Items	N of Items
0.774	0.770	19

As signified, the Cronbach alpha was 0.77, which was on good rating.

To analyze the data, descriptive and inferential statistics were used as it follows:

Descriptive statistics: In this study, descriptive statistics were used to analyze demographic data. For this purpose, demographic data are shown in the frequency table (**Table 2**).

Inferential statistics: for data analysis and hypothesis testing, analytical techniques were used, including: chi-square (X2), regression-test and one-sample binomial test [**3**,**8**,**14**,**16**,**17**].

## Result and Finding

In this analysis, collected demographic data of the sample have been investigated by using appropriate descriptive analysis tools. In this section, the results of descriptive analyzes are presented.

**Table 2:** Frequency table

University education		Age		Sex		FREQUENCY
NO	YES	<40	>40	MALE	FEMALE	
137	163	211	89	162	138	COUNT
45%	54%	70%	29%	54%	46%	%

The results of the questionnaire were noted in **Table 1**. Almost no significant differences between our respondents and the results of the balance will be split equally on both sexes.

The age of the majority of respondents was less than 40 years. In addition, in terms of university education they were educated.

For testing hypotheses (1, 2, and 3) namely, whether between gender, age, education and the rate of adoption of SMS marketing were related, we used the chi-square test. Also correlations between the independent and dependent variable (level of adoption SMS marketing) through regression calculation was done.

Consider the following assumptions:

$$\left\{ \begin{array}{l} H0:\;R=0\\ H0:\;R\neq 0 \end{array}\right.$$

If the hypothesis H0 is rejected, it means that the independent variable on the dependent variable has an effect, and vice versa.

**Table 3:** Chi-square and Regression

Hypothesis	variable Independent	Dependent Independent	chi-square		Regression	
			Asymp. Sig	R	R Square	Adjusted R Square
1	sex	Rate of adoption of SMS Marketing	0.004	0.2	0.04	0.03
2	AGE		0.017	0.07	0.005	0.002
3	University education		0.031	0.087	0.008	0.004

As it was seen in **Table 2**, the decision criteria Asymp. Sig. (2-sided) for the first hypothesis was 0.004, which was less than α = 0/05, a reason to reject the hypothesis H0. According to this, there is a relationship between gender and acceptance of SMS marketing. By considering the response frequency of men with 38% compared to the proportion of women, with 20% interest, showing higher tendency towards SMS marketing.

Decision criteria Asymp. Sig. (2-sided) for the second hypothesis was 0.017, which was less than α = 0/05, a reason to reject the hypothesis H0. According to this, there is a relationship between age and the acceptance of SMS marketing. By considering the response frequency, individuals were older than 40 years with a proportion of 23%. Individuals with a lower age of 40 years, with a 32% interest, showed a low tendency towards SMS marketing.

Decision criteria Asymp. Sig. (2-sided) for the third hypothesis was 0.031, which was less than α = 0/05, a reason to reject the hypothesis H0. According to this, there is a relationship between the university education and the acceptance of SMS marketing. By considering the response frequency, highly educated individuals with 27% proportion presented no high education, with 37% interest showing a low tendency towards SMS marketing.

Testing hypotheses No 4-5-6-7-8-9-10:

We will investigate seven dimensions of research by sample T test.

This study hypothesis test is left-sided so the statistics are -1/ 96. Moreover, the rejection area for the hypothesis H0 statistics is larger than -1/ 96. The percentage error was α = 0/05 and the confidence level was (1-α)0/95. According to the sample size (n = 300), the degrees of freedom are: DF=n-1=299.

The hypothesis test was the following, where x = 3 has been considered:

$$\left\{ \begin{array}{l} H0:\;\mu \geq X\\ H1:\;\mu <X \end{array}\right.$$

Also to confirm the validity of results, each hypothesis will investigate with binomial tests and P = 0.5. Test results are summarized in the following table: 

**Table 4:** Test results

Hypothesis	variable Independent	Dependent Independent	T-test	Binomial tests
			t	P>=3
4	Trust	Rate of adoption of SMS Marketing	2.45	0.63
5	Related information		2.97	0.7
6	Timely information		3.37	0.72
7	Personalization		-2.5	0.55
8	Sending time		-14.37	0.35
9	Frequency		-42	0.29
10	Perceived control		17	0.85

According to the statistic test for the hypothesis no (4, 5, 6), respectively, 2.45, 2.97, 3.37 and the hypothesis number (10) by 17 test statistics are greater than -1/ 96, not being in the critical area, assuming that H0 was acceptable to them. This means on the respondents vision trust, related and timely information, perceived control, all have a positive effect on the adoption of SMS marketing.

These cases were also deduced by a binomial test.

Hypothesis # 7 (personalization), according to the 138 people who answered to this hypothesis as medium, final results of the binomial tests have not accepted. So, assuming H0 was rejected.

Hypothesis # 8 (sending time) had no effect on the rate of adoption of SMS marketing. So, assuming H0 was rejected.

Hypothesis # 9 (repetition and frequency) in addition no effect on the rate of adoption of SMS marketing may be adversely affected regarding brand or company name. So, assuming H0 was rejected.

Results summary

1. Men were more interested in SMS marketing than women.

2. Persons under 40 years old were more interested in SMS marketing.

3. Those without higher education were more interested in SMS marketing.

4. Trust, related and timely information, perceived control, having a positive effect regarding the adoption of SMS marketing.

5. Personalization had no significant positive effect on the amount of adoption of SMS marketing.

6. Sending time had no significant positive effect on the amount of adoption of SMS marketing.

7. Repetition and frequency of SMS marketing had a negative effect on the amount of adoption of SMS marketing.

## Conclusion

1. In the process of study, it was revealed that men are more interested than women in SMS marketing. However, the reason for this tendency was not checked. Now question is: therefore, it should be focused on group of men. Answer is in the meaning of word targeting. If the SMS marketing engage to the needs of women as professional and targeted, can be attracted this segment of population. Using phrases like “remarkable women” or “ladies read” are the type of targeting these large groups of society. Accordingly, topics of interest for both genders in terms of variety and the traction must be identified and duly informed.

2. As the statistical samples were identified, people who were aged less than 40 years had more enthusiasm to accept SMS marketing. If part of this willingness was due to the age of this group, by harmonization between contents and media the issue could be exploited. In case the SMS marketing issues fit the needs of this age group, for example, employment, education, small investments, further audience could be attracted in this marketing method. However, the group who was more than 40 years old, showed less interest at the time the questionnaires were collected, which could also have earned with the concern issues. The identification topics were possible through scientific research, such as buying a house, household commodity basket, discount prices, etc.

3. College-educated of the sample showed less interest in SMS marketing. In this study, the reason for this subject was not investigated. It seemed that those with a higher education probably had better options such as the Internet, social networking for communicating and satisfying their needs by this, using SMS marketing being less attractive for them. In these cases, by providing useful links and adding media files to SMS, a simple message could be transformed into a hypertext page, in addition targeting this group as well as providing technical facilities. But, the enthusiasm of people who were not yet highly educational should not be ignored, because usually the main volume market is not the first and the last deciles, but the medium level makes the mass market [**17**]. Therefore, the needs and interests of these groups have been clearly identified, being the main mobile marketing issue.

4. Recognition and familiarity with the SMS sender was important and was related to an issue (trust) which is one of the main concepts in the business. Companies and successful business keep their relationship with customers via SMS. The concept of CRM (Customer Relationship Management) refers to the set of tasks that involve long-term relationships with companies and customers through strategic relationships, which will lead to the loyalty of consumers and the survival of the company. The use of a dedicated phone number and company name in the text message can help identifying the SMS marketing sender.

5. If the contents of the SMS were synchronized with the interest of the audience, they would have had an effect on the adoption and embrace the SMS advertising. Therefore, one of the main responsibilities of the marketer was to identify the target market needs and develop the marketing list to send SMS with relevant data. Preparing this list was possible through submits on the Internet or during the purchase time through barcodes. Today, barcodes and electronic sales systems can help us to divide the target market. Obviously, the purchase of the other company’s customer lists or the selling of personal information to others or the purchase of customer lists from brokers could reduce public trust and unwillingness of customers to provide their personal information (Marketing Management).

6. Timeliness of SMS marketing had an effect on the rate of adoption of SMS advertising. Usually, in different seasons of year, different events, different times of day, despite irregularities could detect a specific process. People of different gender, ages and cultures, are audiences for advertising, like students, merchants, Grand industry, and artists, etc. In each human case with a variety of drive and motivation, some of the motives that prompted the needs had a higher priority. Assuming that a student needed to find a good grade for holiday or a hungry person looking for a restaurant or investors looking for a trade to provide their products, are all considered priorities. Surely all these people pay more attention to advertising that is tailored to their needs. This issue regarding the today’s community needs, which target the market, have been marked for attention and determine contents of SMS marketing. Bulk SMS sender institutions must have the expert’s team approval to assess subjects until SMS marketing contents coordinate with the needs of the target market.

7. Personalization did not have a significant effect on the adoption to SMS marketing. Personalization has different levels. One level is to address the SMS recipient with name. This level of Personalization usually occurs in companies that have strategic long-term relationships with customers and their relationships have sufficient depth. Another level of personalization is the sending of an SMS based on personal interests and preferences of individuals. This level includes the collection of the consumer information through the questionnaire or barcodes and records them in a database. Personalization is also possible by market segmentation according to the economic and geographical regions and age groups. However, at all levels of personalization refer to audience and contacts mentioned in a certain manner. For example, Mr., Mrs., or athlete honorable, dear neighborhood. As it was clear from the results of the studies personalization, it did not have a high role in SMS marketing. However, this issue depends on the purpose of advertising. Usually, personalization has reversely related to number of SMS.

8. There is no relation between the sending time and the impact on SMS marketing acceptance. Therefore, the sending time is not determined alone. SMS marketing can hopefully be successful with respect to other criteria, irrespective of the sending time. Besides this issue, according to the message content and situation analysis, regarding the risks and opportunities, choosing a time to send a SMS marketing that is not always recommended for audience engagement, should be taken into account. For example, between 9 am and 12 am, for most people it is usually time for work, movement, meetings, etc. The optimum results were achieved when the respondent’s willingness and ability to investigate were simultaneously provided. In addition to the material presented, the purpose of advertising, audience, message content and the problem of time have a considerable impact.

9. The SMS marketing is used in a huge number and can repeatedly face the marketer with reluctant audiences. Due to the limitations in SMS text and graphics, it has a lower diversity and creativity. The repeating of the SMS with a fixed content and form will have a bad effect on audience. Because of the marketing purpose of the SMS, the period of sending the SMS should be more closely controlled to ensure that the capacity of the audience would not escape. Only one sending is ideal. Traditional advertising is protected regarding this subject. Although an ad’s repetition makes it remain in the audience’s memory, unexpected effects should not be ignored. Another issue is related to the tolerance threshold. How many SMS ads can really be read by a person per day? By sending bulk SMS, administrators should consider that, SMS marketing can lead to a disenchantment of audience. What should always be taken into consideration is that the mobile phone is a personal device. According to law and citizenship rights, SMS marketing capacity should be used correctly.

10. There is a relationship between the perceived control and the SMS marketing adoption. The sense of control over the incoming messages can increase the level of its acceptability. Unsubscribing from receiving SMS marketing should go as smooth as possible. For example, sending number 1 to the relevant operator or audience may be reversed after withdrawing and would like to re-join the group receiving. The perceived control has closely related to clients confidence and a positive attitude especially about the personal privacy. Request for permission to freely register to the contacts and the choice for the next posts features are an ethical business. 62% of women and 78% of men were in agreement, which reflects the greater willingness of men to have control over personal belongings.

Future research

This study was a follow-up to an earlier study of the impact of adopting the internet and e-marketing. This would enable future researchers establish the changes that have occurred and the other consequences they have experienced. Some recommended titles are the following:

Identifying interest topics for men and women that by addressing them can increase attractiveness of SMS marketing.

How to identify social trends and ways to use SMS in addressing them?

What are the factors affecting the consumer’s confidence in SMS marketing?

In which status does SMS marketing have a more important role, awareness, persuasion, reminding, or support?

In which stage of the product’s life should SMS marketing be used?
